# Views on ageing: a lifespan perspective

**DOI:** 10.1007/s10433-019-00535-9

**Published:** 2019-10-11

**Authors:** Anna E. Kornadt, Eva-Marie Kessler, Susanne Wurm, Catherine E. Bowen, Martina Gabrian, Verena Klusmann

**Affiliations:** 1 Fakultät für Psychologie und Sportwissenschaft, Differentielle Psychologie und Psychologische Diagnostik, Bielefeld University, Universitätsstraße 25, 33615 Bielefeld, Germany; 2 Department of Psychology, Geropsychology, MSB Medical School Berlin, Siemens Villa, Calandrellistraße 1-9, 12247 Berlin, Germany; 3 Institut für Community Medicine, Abt. für Sozialmedizin und Prävention, Universität Greifswald, Walter-Rathenau-Str. 48, 17475 Greifswald, Germany; 4 Independent Researcher, Vienna, Austria; 5 Independent Researcher, Frankfurt am Main, Germany; 6 Department of Psychology, Psychological Assessment and Health Psychology, University of Konstanz, P.O. Box 47, 78457 Constance, Germany; 7 Department of Psychology and Human Movement Science, Public Health, University of Hamburg, Mollerstr. 10, 20148 Hamburg, Germany

**Keywords:** Views on ageing, Lifespan development, Age stereotypes, Subjective ageing, Self-perceptions of ageing

## Abstract

Views on ageing (VoA) have special relevance for the ageing process by influencing health, well-being, and longevity. Although VoA form early in life, so far, most research has concentrated on how VoA affect later middle-aged and older adults. In this theoretical article, we argue that a lifespan approach is needed in order to more fully understand the origins of VoA, how they change over ontogenetic time, and how they shape development across the full breadth of the lifespan. We begin by explicitly linking VoA to fundamental principles of lifespan development. We review existing theories of VoA and discuss their respective contributions and limitations. We then outline a lifespan approach to VoA that integrates existing theories and addresses some of their limitations. We elaborate on three core propositions of a lifespan approach to VoA: (1) VoA *develop* as the result of a dynamic, ongoing, and complex interaction between biological-evolutionary, psychological, and social-contextual factors; however, the relative importance of different sources changes across the lifespan; (2) VoA *impact* development across the whole lifespan; however, different outcomes, mechanisms, and time frames need to be considered in order to describe and understand their effects; and (3) VoA are *multidimensional, multidirectional, and multifunctional* throughout life, but their complexity, meaning, and adaptivity change across the lifespan. We conclude with recommendations for future lifespan research on VoA.

## Introduction

Views on ageing (VoA) refer to individuals’ conceptions about older people, old age, and ageing in general (such as age stereotypes) as well as conceptions of their own age and ageing (such as self-perceptions of ageing) (Wurm et al. 2017). Many studies have demonstrated that VoA can have far-reaching effects on different indicators of successful development including self-views (Rothermund and Brandtstädter 2003), social integration (Menkin et al. 2017), cognitive functioning (Seidler and Wolff 2017; Siebert et al. 2018), as well as health and mortality (for overviews see Westerhof et al. 2014; Wurm et al. 2017). Based on these findings, it seems safe to say that VoA are an important driver of adult development (Bowen et al. 2014).

In this article, we aim to advance scientific discussion by outlining a lifespan approach to VoA. So far, the primary focus of research has been on how VoA affect middle-aged and older people, given that—at least at first glance—VoA seem most relevant and meaningful for people once they have already become “old” (Levy 2009). However, it seems reasonable to assume that VoA play a powerful role in shaping the course of development across the *entire* lifespan, and not just across the second half. Reciprocally, it also seems reasonable to assume that VoA do not just shape development but are also themselves shaped by people’s experiences as they traverse the life course and transition through different life contexts. We therefore argue that a lifespan approach is needed in order to more fully understand the origins of VoA, how they change over ontogentic time, and how they shape development across the full breadth of the lifespan.

We first draw on fundamental principles of lifespan development to illustrate how VoA reflect a truly lifespan construct. We then discuss existing theories of VoA and consider their contributions as well as their limitations. In order to address some of the gaps in existing theories, we elaborate on three specific propositions that we deem particularly relevant for furthering research on VoA: (1) VoA *develop* as the result of a dynamic, ongoing, and complex interaction between biological-evolutionary, psychological, and social-contextual factors; however, the relative importance of different sources changes across the lifespan; (2) VoA *impact* development across the whole lifespan; however, different outcomes, mechanisms, and time frames need to be considered in order to describe and understand their effects; and (3) VoA are *multidimensional, multidirectional, and multifunctional* throughout life, but their complexity, meaning, and adaptivity changes across the lifespan. We conclude with recommendations for future lifespan research on VoA.

As opposed to detail an all-encompassing framework that covers all VoA constructs, life periods, and developmental outcomes, we aim to provide impulses for future lifespan research on VoA. In order to reduce complexity, we therefore restrict our focus to individuals’ age stereotypes and self-perceptions of ageing about the second half of life. We nevertheless heartily acknowledge the relevance of views on younger people (e.g. Galambos et al. 2005), other VoA constructs (e.g. subjective age; see Barrett and Montepare 2015 and Montepare 2009 for a lifespan approach to subjective age akin to the current article), and within-construct differences (e.g. sub-constructs, specific measures) for a truly complete lifespan approach to VoA. We also acknowledge the relevance of *culturally shared* images of ageing (as opposed to the VoA held by the individual) for lifespan development, which are, however, outside the scope of the current paper.

## Linking views on ageing to fundamental principles of lifespan development

Theories of lifespan development do not generally explicitly address VoA (for exceptions, see Heckhausen et al. 1989; cf. Dutt et al. 2016), but nevertheless provide a helpful framework for demonstrating why VoA are relevant across the entire lifespan. We draw on key principles from the Lifespan Theory of Development (Baltes et al. 2006) and the Two-Process-Model of Developmental Regulation (Brandtstädter 1998) to illustrate why a lifespan approach to VoA is warranted and in fact desirable (Diehl et al. 2014).

### Other people’s VoA represent important social contexts

Lifespan theories of development all agree that contextual factors such as “social others” and their beliefs and opinions influence developmental pathways to a large degree (e.g. Wahl and Gerstorf 2018). Other people’s age stereotypes influence the extent to which they (e.g. an employer, a healthcare professional, a friend or relative) support, constrain, or steer an individual’s development in particular directions. In particular, stereotypes about the limited potential for positive change and learning in the second half of the lifespan can limit older adults’ opportunities for further psychological, physical, or social development. For instance, healthcare providers have traditionally assumed that there is little potential for health improvements in old age. Accordingly, older adults have often been denied access to adequate and high-quality treatment, e.g. regarding psychotherapeutic care (Kessler and Schneider 2017; Kessler and Blachetta 2018). Similarly, in the workforce older employees often face age stereotypes about their (limited) potential to learn and master change, which in turn limits their opportunities for career growth and further training (Bowen and Staudinger 2013; Chui et al. 2001; Van Daalen et al. 2010).

Far less research has focused on the effects of other people’s potential-oriented VoA, but there is evidence that other people’s positively valenced VoA can foster older people’s functioning. For instance, older people’s cognitive performance increases after discussing a difficult life situation with an adolescent—a social context which likely activates the stereotype about older people’s wisdom and older people’s motivation to pass on knowledge to the younger generation (Kessler and Staudinger 2007). Another study found that age was unrelated to the motivation to strive towards advancement and growth when employees believe that older workers are perceived more positively in their company (the motivation to strive towards advancement typically declines with age; Bowen and Staudinger 2013).

In sum, other people’s VoA influence individuals’ experiences and the resources they are able to access in different social contexts. Importantly, other people’s VoA also affect younger people: observing older people in particular social roles (Bowen and Skirbekk 2013) and observing how they are treated in different social contexts shape younger people’s expectations for their own future, and thus impact the direction of their development.

### Individuals’ own VoA guide self-directed development

Lifespan theories of development also acknowledge that people play an active role in guiding their own development (e.g. Brandtstädter 1998; Lerner 1984). Across the whole lifespan, VoA help to form one’s ideas about the life one could live and the person one could be as one ages. Individuals’ own age stereotypes and ageing self-perceptions shape their expectations and possible selves and thus inform their goals and action selection (Kornadt and Rothermund 2012; Lloyd et al. 2018; Rothermund 2005) and also their beliefs about personal agency and self-efficacy (e.g. Klusmann et al. 2019b; Levy 1996; Wurm et al. 2013). As a concrete example, people who perceive their own future as an older person in a more positive light report preparing more for anticipated age-related changes, since they believe that they can shape their development accordingly (Kornadt et al. 2015). Although most research has focused on how VoA affect self-directed development specifically in later life, together with some further evidence the study of Kornadt and colleagues also indicates that age stereotypes and ageing self-perceptions affect self-directed development already at younger ages (see also Bowen et al. 2019; Klusmann et al. 2019b).

### VoA are embedded within a greater historical, cultural, and societal context

Another cornerstone of lifespan development is recognition of the fact that people are embedded in historical, cultural, and societal contexts which both create and limit their developmental opportunities. Understanding how VoA themselves develop and operate likewise necessitates consideration of (changing) historical, cultural, and societal conditions. Normative social roles, life course scripts, and age-graded institutions (e.g. retirement practices) represent culturally shared expectations about development and also whether behaviours and events are perceived as “appropriate” or “on time” (Neugarten et al. 1965; Settersten and Hagestad 2015). The adherence to these expectations is rewarded and non-adherence is punished, and thus, societal expectations influence the extent to which individuals have opportunities and leeway for behaviour at any given age. The characteristics ascribed to older people and old age have also been shown to change over time, as shown for example using data from the German Ageing Survey (Wurm and Huxhold 2012) or linguistic analysis of 200 years of printed texts in the USA (Ng et al. 2015). Changes in VoA have been linked to historical forces such as population ageing (Wolff et al. 2018) or the youth-centeredness of a culture at a given historical time (e.g. Westerhof et al. 2011). Relatedly, the consequences of VoA at different stages of the lifespan need to be interpreted and understood against the backdrop of the particular historical, cultural, and societal contexts in which they operate (cf. Wahl and Gerstorf 2018). For example, a fixed retirement age in a society might serve as a salient marker of becoming “old” and thus trigger the impact of related VoA (Kornadt et al. 2017). However, we have to note that with exceptions, research on VoA has been conducted mostly in Western countries, and thus, our knowledge of its cultural aspects is still limited.

### VoA are multidimensional, multidirectional, and multifunctional

Just as lifespan development consists of both gains and losses in different domains of functioning (Baltes et al. 2006), VoA are likewise multidimensional and multidirectional (Diehl et al. 2014; Kornadt and Rothermund 2015; Wurm et al. 2017). Research has tended to focus on the adverse impact that age stereotypes and self-perceptions of ageing concerning the limitations of old age can have on older people’s functioning and well-being. On the whole, people appear to perceive that older age is characterized by more losses than gains (e.g. Heckhausen et al. 1989) and negatively valenced VoA about older people’s deficits appear to be rather common (Kite et al. 2005). However, a large body of evidence has demonstrated that people actually have many different VoA about older people’s relative advantages and disadvantages, which, for instance, vary across life domains (Kornadt and Rothermund 2011, 2012). Although the literature has often tended to focus on the ageing-related deficits and losses, when people think about old age and ageing they also think about a number of strengths and gains such as wisdom (Heckhausen et al. 1989; Montepare et al. 2014), reliability (Posthuma and Campion 2009), and warmth (Cuddy et al. 2005). People also have many VoA which cannot be easily organized along a simple positive–negative dimension. For instance, it was shown that people have views of different “types” of older people (e.g. “the Curmudgeon” or “the Golden Ager”; Hummert et al. 1994). People are also more or less aware of their own ageing in different contexts (Miche et al. 2014b), and VoA emphasize different aspects of ageing, depending on the life domains (Klusmann 2019). Not only are they multidirectional and multidimensional, VoA are also *multifunctiona*l: for example, believing that particular social or health losses are an inherent part of getting older may offer some emotional comfort in the face of loneliness or a negative health event, but also detrimental with regard to motivating efforts to change the situation. The same VoA may thus have both adverse and beneficial effects within and across domains. Hence, the “adaptivity” of VoA must be viewed relative to a specific outcome (e.g. domain of functioning, time frame) and context (e.g. life phase, opportunities, and constraints imposed by the environment).

### VoA link a person’s past, present, and future

Finally, a lifespan approach to VoA is warranted given that VoA reflect a person’s anticipation of the future as well as their understanding of their present and past. In youth, VoA primarily reflect representations of an “outgroup” and abstract notions of the distant future. As people near midlife, individuals increasingly interpret and evaluate their experiences as being “age-related”, and their VoA begin to refer to themselves and their peers, and thus increasingly include representations of the present. As they approach advanced old age, people’s VoA become more and more entangled with their understanding of their own past. Hence, as people proceed through the life course, their ontogenetic experiences (e.g. social exchange in difference age-graded contexts, physical changes) also shape their understanding of what it means to become older and to be old (e.g. Rothermund and Brandtstädter 2003; Wurm and Huxhold 2012).

### Summing up

**Fig. 1:**
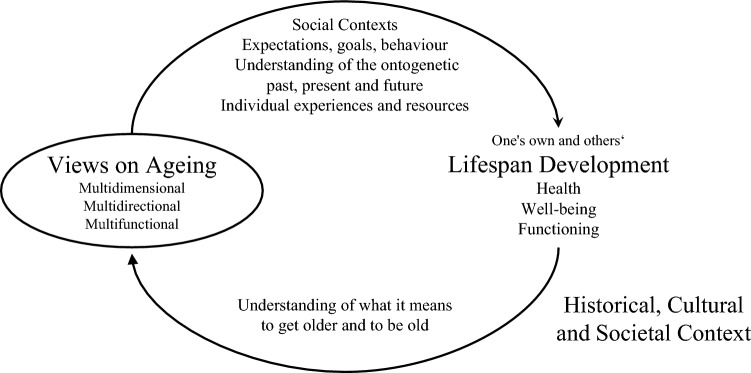
Views on ageing as both drivers and products of lifespan development

Given the close links between VoA and the principles of lifespan development, a lifespan approach is not only warranted but inevitable in order to fully understand the content and characteristics of VoA, how VoA develop over ontogenetic time, and how VoA impact development. Figure 1 illustrates VoA as both *drivers* and *products* of development across the entire lifespan (Bowen et al. 2014): for better and for worse, VoA affect development (i.e. outcomes such as health, well-being, and functioning) by affecting people’s social contexts, their self-development via their expectations, goals and behaviour, their mental models of their own past, present and future, and their individual experiences and resources. Reciprocally, how individuals develop over the lifespan shapes their understanding of what it means to age and be old in different life domains, and hence revise their VoA.

## Existing theoretical frameworks of views on ageing

*Stereotype Embodiment Theory* (SET, Levy 2003, 2009), *Terror Management Theory* (TMT, e.g. Martens et al. 2005), and *Awareness of Age*-*Related Change* (AARC, Diehl and Wahl 2010) are three prominent theoretical approaches that have previously dealt with the development, impact, and characteristics of VoA. The three approaches differ substantially with regard to their scope and predictions, and none provides a complete framework for understanding VoA across the lifespan.

The basic idea of SET is that people develop old-age stereotypes early in life based on their interactions with older persons and exposure to (negatively valenced) culturally shared images of ageing communicated by, for example, children’s books and other media (Levy 2009). Young people are assumed to internalize culturally shared images of ageing more or less unquestioned because they are not self-relevant, and because there may even be a number of benefits for valorizing their own young age group at the expense of older adults. Then, as people grow older and begin to self-identify as “old”, their age stereotypes turn into self-fulfilling prophecies, affecting health and well-being via physiological (e.g. stress responses), psychological (e.g. will to live), and behavioural (e.g. health behaviour) mechanisms (Levy 2009).

In contrast to SET, TMT focuses on the role of evolutionary-based fear of death, rather than the internalization of culturally shared images of ageing as the root of VoA that devalue older adults. According to TMT, older people function as a reminder of transience and mortality. VoA that emphasize older people’s deficits are therefore assumed to arise as a mechanism for dealing with the fear of death and senescence by creating psychological distance between oneself and the group of “older people” (Martens et al. 2005). Accordingly, deficit-oriented VoA are thought to serve as an anxiety-buffer, helping people to protect their sense of self-esteem, worthiness, and sustainability despite the awareness of the inevitability of death (Martens et al. 2004).

Finally, AARC refers to the cognitive awareness of how one is changing with chronological age. According to AARC theory, awareness that one is growing older becomes represented as a specific facet of one’s self-knowledge and ultimately turns into a central part of a person’s overall self-representation (Diehl et al. 2014). AARC assumes that people become aware of their own ageing as they (a) perceive that they are changing or have changed (for the better and for the worse) and (b) associate such changes with the ageing process. AARC suggests that the salience of and/or sensitivity to “age cues” begins primarily in midlife and increases with age. AARC rests on the idea that VoA are multidimensional and domain-specific (the authors identify the key domains of health and physical functioning, cognitive functioning, interpersonal relations, social-cognitive and social-emotional functioning, as well as lifestyle and engagement). AARC also underlines the outstanding role of social context, and social role transitions are thought to act as important reminders of a person’s position in life (Miche et al. 2014b).

### Contributions and limitations of existing theories

SET, TMT, and AARC have made a number of contributions to our understanding of the characteristics of VoA, how VoA change across the lifespan, and how VoA impact development. Integrating the three theories, VoA are thought to develop already in childhood but increase in salience with older chronological age, explain why people tend to have rather negatively valenced VoA about the second half of life and distance themselves from the group of “older people”, and finally how VoA affect development through a number of physiological, psychological, and behavioural mechanisms. However, existing theories also have a number of gaps. Neither SET nor TMT explicitly acknowledge people’s experiences with their own ageing as a source of their VoA (Miche et al. 2014a; Kornadt et al. 2017; Rothermund and Brandtstädter 2003). AARC focuses on how VoA develop only in the second half of life; relatedly, SET focuses on the impact of VoA only in the second half of life once VoA become self-relevant. TMT’s explanation for why negatively valenced VoA should prevail across life does not sit well with evidence that death anxiety is in fact lower in old age relative to other life phases (e.g. Russac et al. 2007), or that older adults do not respond to reminders of their mortality in the same way than younger persons do (e.g. Maxfield et al. 2014; Bluntschli et al. 2018). SET does acknowledge that VoA differ in content (e.g. age stereotypes about cognitive and physical domains) and this impacts their influence on developmental outcomes (e.g. cognitive and physical outcomes, respectively; Levy and Leifheit-Limson 2009), but only AARC explicitly acknowledges that VoA are multidirectional (i.e. AARC includes representations of age-related gains) as well as multidimensional (i.e. as opposed to organized along a global positive–negative dichotomy). Thus, so far, no existing theory represents a comprehensive lifespan approach to VoA that adequately takes into account the multidimensionality, multidirectionality, and multifunctionality of VoA, as well as the developmental dynamics of VoA across the *entire* lifespan.

## Three core propositions of a lifespan approach to views on ageing

To address some of the gaps in existing theories of VoA, we now draw on existing lifespan developmental and VoA theories as well as empirical evidence to elaborate on three core propositions of a lifespan approach to VoA (see Table 1 for a summary). We believe that research regarding these three core propositions in particular would substantially advance our understanding of VoA as drivers and products of development across the entire lifespan. Again, our intent here is to stimulate discussion and push VoA research in new directions as opposed to provide final answers or a fully comprehensive theory.

**Table 1:** Summary of propositions and empirical findings

Question	What shapes VoA across the lifespan?	What is the impact of VoA across the lifespan?	What characterizes VoA across the lifespan?
Proposition	1. VoA develop as the result of a dynamic, ongoing and complex interaction between biological-evolutionary, psychological, and societal-contextual factors; however, the relative impact of different sources changes across the lifespan	2. VoA impact development across the whole lifespan; however, different outcomes, mechanisms, and time frames need to be considered in order to describe and understand their effects	3. VoA are multidimensional, multidirectional, and multifunctional throughout life, but the complexity, meaning, and adaptivity of different VoA changes across the lifespan
Findings	Three sources of individual VoA (bio-psycho-social) interacting and mutually influencing each other throughout life	Long- and short-term impact of VoA at all life stages; cumulative effects across life	Increases in complexity as people get older; possible decreases in oldest age
	Sources with special relevance for *Childhood through young adulthood*	Important outcomes in *childhood through young adulthood*	Drivers of complexity dependent on life stage
	Media	Self-perceptions	
	Intergenerational contact	Anxiety	Complexity as a self-reinforcing process throughout life
	Social learning	Risk and health behaviours	
	Ageing anxiety/Terror Management	Opportunities in the workplace	
	Resource conflicts		Adaptivity of VoA dependent on context and content
		Important outcomes *at the end of life*	
	*Middle adulthood*	Late-life health issues	
	Age experiences in the workplace	Gerotranscendence	Complex and multifaceted representations of ageing and old age provide highest adaptive potential
	Ageing anxiety/Terror Management	Ego-Integrity	
	Family dynamics		
	Physical changes		
		Physiological, psychological, and behavioural pathways change in relative relevance across life	
	*Older adulthood*		
	Role transitions into “old” roles		
	Ageing social networks		
	Loss of physical functioning		
	Couple and gender dynamics		
	Personality traits		
	Experiences of ageism		
	*Proximity to death*		
	Age-attributed health decline		
	Environmental contexts		
	Generativity/transcendence		

### **Proposition 1**

***VoA develop as the result of a dynamic, ongoing, and complex interaction between biological***-***evolutionary, psychological, and societal***-***contextual factors; however, the relative impact of different sources changes across the lifespan.***

Throughout the lifespan, we propose that there are three sources of VoA—biological-evolutionary, psychological, and social-contextual—that interact and mutually influence each other. *Biological*-*evolutionary* sources include genetic influences (Kornadt and Kandler 2017), bodily signs of ageing as well as changes in physical functioning, and the evolutionary aspects reflected in terror management. *Psychological* sources include basic personality traits such as the Big Five (e.g. Allen et al. 2015; Kornadt et al. 2019; Miche et al. 2014a), or control beliefs (Bellingtier and Neupert 2019). *Societal*-*contextual factors* include factors on the macro-, meso-, and micro-levels such as population ageing, (shifting) culturally shared images of ageing, cultural values, media images of older people, other people’s VoA in the work, and healthcare context, specific role models and relationships with older people, the observation of ageing processes in others, and idiosyncratic experiences with older adults in general. Furthermore, gender roles may also provide a context which contributes to the formation and development of VoA throughout life (Settersten and Hagestad 2015).

### Lifespan dynamics

While the interaction between biological-evolutionary, psychological, and societal-contextual sources is responsible for many, if not most, psychological constructs, VoA are by definition inherently intertwined with individual ageing and the (age-related) experiences in all three domains that people make as they get older. Thus, in order to understand how VoA themselves develop across ontogenetic time, we argue that it is also necessary to consider how the relative influence of the respective sources may change across ontogenetic time.

In line with SET, we expect that VoA in *childhood and adolescence* are most strongly related to societal-contextual factors, such as the culturally shared images of older people communicated by the media, but also information about ageing that is endorsed and transported by parents, peers, and grandparents (Lineweaver et al. 2017). Children’s relationships with older adults (e.g. their grandparents) also likely play a crucial role (Flamion et al. 2017; Pinquart et al. 2000), which might also be shaped by the larger historical, cultural, and societal context children grow up in (i.e. the number of older people in their region or country; cf., Wolff et al. 2018). One’s own experiences of getting older do not play a relevant role yet, but as soon as individuals realize that their own life and that of loved ones is finite, presumably, death-associated anxiety starts to evolve and terror management processes become relevant (e.g. Wisdom et al. 2014).

In *young adulthood*, negatively valenced VoA may arise because old age is often associated with diminished autonomy and control, which contrasts with the developmental tasks of young adulthood (Weltzien et al. 2014; see Galambos et al. 2005 with a related analysis on subjective age). Intergenerational resource conflicts within a society likely become a source of VoA as people enter the workforce and begin paying taxes (North and Fiske 2012), and we expect that contextual factors and experiences related to the workplace become significant sources of VoA sometime between young adulthood and midlife (Bowen et al. 2011). Please note that so far most of the aforementioned studies are about age stereotypes, and few studies have investigated the origins of self-perceptions of ageing in adolescence and young adulthood (for exceptions, see Packer and Chasteen 2006; Patterson et al. 2009).

Throughout *midlife*, we would expect to find a gradual increase in the relative importance of age-related experiences in different life domains, consistent with the concept of AARC. In general, in midlife gradual biological ageing processes do not result in functional limitations yet, but are starting to be noticed (and attributed to age). Different biographical experiences that individuals attribute to ageing might contribute to increasing interindividual variance in VoA, especially for interindividual variation in positive conceptions of old persons (Kornadt and Kandler 2017), but also for ageing self-perceptions. The proximity of becoming a member of the older age group in midlife likely gives further rise to age-related fears, resulting in a stronger devaluation of and dissociation from old age in the sense of TMT (e.g. Kite and Wagner 2002; Kornadt et al. 2016; Weiss and Kornadt 2018). Another, thus far unexplored source of VoA in middle age is rooted in family dynamics. Here, an “empty nest” as well as the ageing and eventual loss of one’s parents or close relatives would seem to be influential experiences (cf. Kim et al. 2018). For women, menopause as a biological age marker seems meaningful as a marker of becoming old (Heckhausen et al. 2001).

In *young*–*old age*, basic personality traits and control beliefs seem to become more important sources of VoA than in earlier life phases, possibly via their influence on the acquisition of experiences (Allen et al. 2015; Bellingtier and Neupert 2019; Kornadt et al. 2019; Miche et al. 2014a). Markers of old age, such as being retired or becoming a grandparent, become more prevalent, and more difficult to ignore and thus stimulate individuals to actively confront and renegotiate their VoA, on their own and also in their social networks and partnerships. Given that social roles and expectations during the transition to becoming “older people” have been different for older men than for older women, there might be some gendered aspects with regard to how age-related experiences in contexts like work and family influence VoA (Lössbroek and Radl 2018). However, as people approach old age, gender differences are likely to decrease as the differentiation between “male” and “female” roles becomes more flexible (Settersten and Hagestad 2015). Normative age-related biological losses together with major and minor health events and, in particular, resulting functional impairment become individual experiences that are attributed to ageing. These experiences might impact how one sees one’s present status and one’s future as an ageing person, but also how one views older people in general (Kornadt et al. 2017; Wurm et al. 2019). With regard to interpersonal processes, dyadic processes in ageing couples can be observed with partners’ VoA reciprocally influencing each other (Kim et al. 2018; Mejía and Gonzalez 2017). Experiences of age discrimination might also become a more important source of VoA in young–old age; this is particularly true for self-perceptions of ageing once a person begins to be identified as “old” by others (Hooker et al. 2019).

In *old*–*old age*, we assume that ontogenetic experiences in the health domain become the most important source of VoA. Which other sources (if any) influence, VoA would seem to be strongly dependent on the individual’s health in this final phase of life. There is some evidence that self-perceptions of ageing universally (i.e. with little interindividual variation) become more pessimistic as people get older (Miche et al. 2014a). Importantly, however, we assume that the effect of health experiences is moderated by contextual factors, such as the “age-friendliness” of one’s neighbourhood (e.g. quality of public transportation, accessibility to shops and health care; Wahl and Gerstorf 2018; Wolff et al. 2018; Wurm et al. 2014). Gender would seem to be an important developmental context again in old–old age, given women’s higher life expectancy: “to be very old, is to be female” (Settersten and Hagestad 2015, p. 31). Generativity and death issues also become paramount and by highlighting the finitude of life, they likely influence VoA.

#### **Proposition 2**

***VoA impact development across the whole lifespan; however, different outcomes, mechanisms, and time frames need to be considered in order to describe and understand their effects.***

We propose that VoA exert their long- and short-term impact at all life stages. Since midlife and young–old age have been extensively covered, we will concentrate on how VoA affect younger people (i.e. people under age 30 or 40) and the oldest-old (i.e. people 80+ years), and how the effects of VoA on younger people and the oldest-old may be better revealed by considering particular outcomes and time frames. We also touch upon how the mechanisms by which VoA affect development may vary across the lifespan.

### The disregarded impact of VoA on younger people

Although few studies have investigated how VoA concerning older people may impact younger people, there is some evidence that VoA have a number of short- and long-term effects also in the first half of the lifespan. Other people’s VoA about older people’s deficits may in some cases benefit younger people, such as when younger employees are provided more opportunities for career advancement or further training (Martin et al. 2014). In contrast, other people’s VoA about older people’s higher (compared to younger people) reliability and accuracy (van Daalen et al. 2010) might impede young people’s ability to secure paid work.

There is also some theoretical reasoning that younger people’s VoA concerning the second half of the lifespan impact their mental models of their own development already in younger years (e.g. Baranowski 1982; Galambos et al. 2005; Gilbert and Ricketts 2008; Seefeldt et al. 1981). Empirical evidence linking VoA and young people’s mental models of their own future, however, is scarce (for a cross-sectional exception, see Lloyd et al. 2018), and there is also evidence that younger people’s old-age stereotypes and their self-perceptions are unrelated (e.g. Newman et al. 1997; Patterson et al. 2009). It is also often presumed that younger people’s age stereotypes are related to their personal ageing anxiety, though the relationship has rarely been tested (Montepare and Zebrowitz 2002; for an exception, see McGuinn and Mosher-Ashley 2002). Based on the results of cross-sectional studies, some authors have suggested that young adults engage in risky behaviour, such as sexual risk behaviours and illegal drug use as a specific form of terror management, that is, to fight death anxiety by feeling invulnerable (e.g. Hughes et al. 2016; Kennison and Ponce-Garcia 2012; Popham et al. 2011a, b). Young adults with more overall positive VoA, in contrast, may avoid taking risks because they wish to live as long as possible (Popham et al. 2011b). Similarly, young adults with more positive and less negative VoA indicate a preference to live longer (Bowen and Skirbekk 2017), and young adults who want to live longer are also less likely to smoke or be physically inactive—presumably because they are more motivated to invest in behaviours that may extend their lifetimes (Bowen et al. 2019).

### The impact of VoA at the end of life

VoA are linked to some of the most prevalent health issues in the last phase of life, such as dementia and Alzheimer’s disease (Levy et al. 2016a, 2018), frailty (Gale and Cooper 2018), or even mortality (e.g. Kotter-Grühn et al. 2009; Levy et al. 2002; Sargent-Cox et al. 2014). Thus, VoA seem to be involved in processes leading to these late-life developmental outcomes.

Importantly, the maintenance of a certain status quo could become more difficult as one approaches the end of life (Baltes and Smith 2003; Kotter-Grühn et al. 2009), and thus, overly optimistic VoA about one’s potential for continued good health and functioning might be maladapative when gains and/or maintenance are no longer realistic. Capturing the effect of VoA on late-life development and assessing the adaptivity of particular VoA in late life might thus be better addressed with regard to outcomes other than health status or conventional well-being measures. For example, relevant end-of-life values (e.g. gerotranscendence; Brandtstädter et al. 2010) or mastering the developmental tasks of late life (e.g. integrity) might be more suitable outcomes to address.

### The shifting relevance of different mechanisms

Theoretically, the same behavioural, physiological, and psychological mechanisms by which VoA influence developmental outcomes in later life could apply to younger adults as well, though their effects may be much subtler and/or only be revealed over time. However, since most studies include only midlife and older participants, it is still far from clear whether VoA actually operate via the same behavioural, physiological, and psychological pathways (Levy 2009) across all life stages, and/or how the relevance of particular mechanisms may shift over ontogenetic time.

With regard to social-cognitive mechanisms, Klusmann et al. (2019b) recently demonstrated that younger adults (18–35 years) with more positive self-perceptions of ageing at baseline improved their eating behaviour over time. The effect of VoA on eating behaviour was, however, larger in older age groups due to a stronger productive social-cognitive dynamic (e.g. higher self-efficacy). Interestingly, despite well-known gender differences in healthy eating behaviours, there were no gender differences regarding the mechanisms by which VoA impacted young men and women’s eating behaviour. With regard to physiological mechanisms, Levy et al. (2016b) showed that self-perceptions of ageing longitudinally impacted cortisol levels in older, but not in younger people. It might thus be possible that physiological mechanisms such as the effects of VoA on the hormonal stress response and cardiovascular reactivity increase towards the end of life. VoA might affect younger people’s development more via anxiety but older people’s development via their effects on the self-concept. Besides, we would expect future expectations to be a more important psychological pathway to explain the effect of VoA on younger relative to older adults.

### Considering different time frames

Considering long time spans is particularly relevant for revealing the effects of VoA held earlier in life on health-related outcomes later in life. For instance, in a well-cited study, Levy et al. (2009) found that people who had negative-age stereotypes in early and mid-adulthood (age 18–49) had a substantially increased risk for cardiovascular events in old age, up to 38 years later. Still, VoA can be a motor for development in the short as well as the long-run for people of all ages. As an example, a younger person’s VoA might motivate him or her to spend more quality time with older people, with the short-term benefit of potentially being able to profit from older people’s life experience and the long-term benefit of potentially developing diverse and differentiated attitudes towards their own ageing by having had more contact with older people (e.g. Jarrott and Savla 2016; Kotter-Grühn 2015). This assumption of short- and long-term impact of VoA across life corresponds to the reasoning of Westerhof and Wurm (2015, 2018) who concluded that the beneficial effects of potential-oriented VoA held early in life on later life health result from an accumulation of psychological and behavioural resources across the lifespan.

#### **Proposition 3**

***VoA are multidimensional, multidirectional, and multifunctional throughout life, but the complexity, meaning, and adaptivity of different VoA changes across the lifespan.***

As previously described, a number of studies have demonstrated that VoA are inherently complex. We propose that the complexity of VoA is one central feature necessary to be considered for their lifespan understanding. There are still many open questions regarding the complexity of VoA, including what determines the development of complexity, whether more complex VoA are generally more adaptive and whether the adaptivity of more complex VoA changes across the lifespan.

### The development of complexity

There is evidence that even children as young as 5 years old have multidimensional VoA, and that VoA continue to be multidimensional throughout childhood, adolescence and young adulthood (Hummert et al. 1994; Jarrott and Savla 2016; Lloyd et al. 2018; Mitchell et al. 1985; Montepare and Zebrowitz 2002; Schmidt-Hertha et al. 2012). Hummert et al. (1994) reported that older persons had somewhat more differentiated prototypes of older adults than their younger and middle-aged counterparts. A very recent study compared photographs taken by younger (age 20–30), young–old (age 50–69), and old–old participants (aged 70 plus) as an alternative method for capturing their VoA (Klusmann 2019). Besides differing somewhat in content, older adults’ pictures were only slightly more differentiated and less positive than the pictures taken by the young and middle-aged participants. We are unaware of any studies that have compared age differences in other existing indicators of complexity (e.g. dimensionality), and there is little evidence with regard to how the complexity of VoA might *change* across the lifespan.

We would argue that diverse experiences and differentiated knowledge about ageing and old age are likely to contribute to the expression and/or development of more complex VoA throughout life (cf. Heckhausen et al. 1989; Kornadt and Rothermund 2011). However, the drivers of complexity are likely to depend also on the life stage in question. In old–old age, for example, shifts in developmental tasks and value orientations, such as the increase in generative and ego-transcending values (e.g. Brandtstädter et al. 2010) may add further dimensions to VoA that are not present in earlier phases of the lifespan. On the contrary, it is also possible, however, that death-related concerns, loss of autonomy, and health deterioration at the very end of life might limit the focus and complexity of VoA, resulting in rather unidimensional (deficit- and loss-oriented) VoA.

Besides, VoA in different dimensions are not completely independent: perceiving age-related gains or losses in one domain (e.g. such as finances) can “spread” to other, related areas of life (e.g. leisure; Kornadt et al. 2017). The interaction between VoA in different life domains adds an interesting aspect to consider in future research on the differentiation of VoA across the lifespan.

Finally, we propose that complexity is a self-reinforcing process: complex VoA increase the likelihood of behaviour and cognitive appraisals that further enhance the complexity of VoA. In contrast, people who avoid thinking about old age and ageing or have little meaningful contact with older people probably having less elaborated VoA. In some cases, however, repeated negative contact with older people in particular contexts might reinforce one-sided VoA (e.g. care workers primarily exposed to older people with health problems, functional limitations and/or a high need for assistance in daily living, see also Kessler et al. 2014).

### How does complexity relate to the adaptivity of VoA?

Findings based on unidimensional conceptualizations of VoA suggest that priming “negative” (i.e. deficit-oriented) VoA seem to have a larger impact on older people’s behaviour in experimental settings than priming “positive” (i.e. potential-oriented) VoA (Meisner 2012). It is unclear, however, to what extent the same might also be true with regard to positive and negative VoA outside of the laboratory and with regard to developmental outcomes. Furthermore, when and how VoA—irrespective of their valence—are most influential and adaptive depends on the greater context, including life stage, life domain and corresponding outcome variable. For instance, Kornadt et al. (2015, 2017) found in two studies based on a large sample of adults aged 30 to 80 years that the impact of VoA in different life domains on how people see themselves and their preparation for age-related changes depended on the age-dependent relevance of the life domain (i.e. fitness and appearance for the 30–50 years old, work for participants aged between 50 and 65, and social relations for the oldest participants, aged 65 to 80). As a second example, Wolff et al. (2017) examined the impact of ageing self-perceptions on how people coped with a serious health event. Compared with their more optimistic peers, they found that people who more strongly associated their own ageing with health declines had less negative affect six months after experiencing a serious health event, but also more functional limitations two and a half years later. Presumably, associating ageing with health declines helped to *emotionally* prepare participants for a negative health event, but also undermined their motivation to *actively invest* in their physical recovery. The study thus nicely demonstrates that even deficit-oriented VoA serve an important function, and that the same VoA can be considered adaptive and maladaptive depending on the context and particular outcome considered.

More research is strongly needed to clarify whether the match between VoA-domain and outcome-domain increases their impact. This was, for example, posited in the stereotype-matching hypothesis (Levy and Leifheit-Limson 2009) as well as by domain-specific approaches (Kornadt et al. 2017). Second, it has to be determined whether the degree of complexity of VoA itself is adaptive. In the long-run, it would seem that a complex and multifaceted representation of old age might be best for mastering challenges and developmental tasks throughout life, similar to findings regarding self-concept differentiation (e.g. Diehl et al. 2001). For example, people with one-sided, deficit-oriented VoA are unlikely to productively deal with age-related changes, while people with one-sided, overly positive VoA may find themselves unprepared to cope with the challenges of ageing (e.g. a parent’s need for assistance, one’s own declining health, or loss of a youthful appearance). Hence, we argue that research based on more complex conceptualizations of VoA and more nuanced measurement approaches would allow for a deeper understanding of VoA across the lifespan. Unidimensional approaches might be helpful, however, for examining the relationship between VoA and broad, global outcomes. As we have recently found, however, unidimensional and global VoA measures can hardly be empirically differentiated from general dispositions such as optimism (Spuling et al. 2019).

## Recommendations for future research on VoA

Finally, we turn our attention to what is in our opinion still needed to take lifespan research on VoA to the next level. Again, instead of being exhaustive, we will briefly elaborate on points related to the measurement, construct validity, study designs, and conceptualization of VoA across life.

### Establish ways to measure and quantify the complexity of VoA

We have argued that the complexity of VoA is highly relevant. However, there is currently a lack of available indicators and measures of VoA complexity. Furthermore, most available data on VoA rely on questionnaires and other self-reports. Although many existing measures are well-validated and helpful, these might not fully capture all aspects of VoA needed for a truly profound lifespan understanding; it seems time to consider additionally considering and developing more qualitative and/or implicit measures (see Klusmann et al. 2019a).

### The meaning of different VoA needs to be established across age groups

In order to be able to compare different VoA across age groups, we need to understand what different VoA “mean” for different age groups. In younger years, for instance, age stereotypes can be assumed as being similar to stereotypes about other outgroups (such as the opposite gender, or other nationalities), and, generally, young people want to become older (Galambos et al. 2005; Montepare 2009). Thus, when asked about ageing and/or older people, a younger person might be thinking about life in the 40 s or 50 s, whereas a middle-aged adult might have life at a much older age in mind. When they think about getting older, younger people may be thinking about more immediate changes as they transition to young adulthood and midlife (e.g. gaining independence, earning more money) as opposed to becoming a young–old or old–old person. Thus, it might well be that stereotypes about older persons in general and self-perceptions of ageing are completely distinct constructs throughout adolescence and emerging adulthood. Later in life, however, as people begin to self-identify as “older”, age stereotypes and self-perceptions are assumed to converge and become conceptually (and empirically) more similar (e.g. Kornadt et al. 2017). Awareness of this problem and a better understanding about the co-development of age stereotypes and self-perceptions is strongly needed to enable valid implications from age-comparative and longitudinal studies.

### Understand how VoA are related to other psychological constructs

We also need to better understand the distinctiveness of VoA relative to other psychological constructs that are not defined by their age-relatedness but likewise influence developmental regulation, such as control beliefs, self-efficacy, or general optimism. The relation of VoA to these variables may likewise change across the lifespan (see Spuling et al. 2019). Thus, knowledge of dynamic relationships between the variables across life would help to sort VoA into a broader personality system (e.g. Hooker and McAdams 2003; Rupprecht et al. 2019).

### Gather longitudinal data from lifespan samples

Even though several large, longitudinal datasets contain information on age stereotypes, self-perceptions of ageing, or (rarely) even both, they are usually confined to just middle-aged and older adults. There is a need of longitudinal datasets that also include children’s and adolescents’ VoA. This would allow researchers to adequately address how (the sources of) VoA change across the lifespan, issues related to the validity of different VoA constructs in different age groups as described above, and investigate how VoA may also affect younger people. Modern technologies and analyses for micro-longitudinal assessments would allow for new insights into the fluctuation and processes of VoA, capturing their dynamics across life (e.g. Bellingtier and Neupert 2018a, b; Schmidt et al. 2018).

### Assume that all VoA have costs as well as benefits

To date most research has at least implicitly assumed that VoA concerning the deficits of old age and the losses associated with ageing have adverse effects, while VoA concerning the advantages of old age and gains associated with ageing will have beneficial effects. We feel that this sort of thinking has greatly limited our understanding of the full effect of VoA on development and has also prevented our understanding of how we can encourage people to develop more adaptive VoA. We therefore encourage researchers to consider the multifunctional nature of VoA and try to more fully understand and assess their costs and their benefits which may only become apparent when considering multiple time frames and life domains.

### Avoid talking about VoA as “positive” or “negative” as much as possible

While it has advantages in the sense of parsimony and simplification, we also feel that categorizing and referring to VoA as either “positive” or “negative” is in many cases overly simplistic and insufficient. Describing particular VoA depending on their valence also increases the risk of interpreting them as “positive = good” and “negative = bad”, respectively. As we have demonstrated above, VoA that one-sidedly emphasize the gains, strengths and potentials of old age/ageing might be counterproductive in different regards for example whenever they obstruct self-care or coping with contextual obstacles or complaints. Talking about VoA that emphasize older people’s strengths and the gains associated with ageing as “positive” also comes with the risk of overlooking that such VoA emphasize the disadvantages and deficits of younger adults. We therefore heartily encourage researchers to think of other, more precise ways of describing VoA, such as potential- and deficit-oriented, or gain- and loss-related VoA.

## Conclusion

VoA are both drivers as well as products of development across the entire lifespan, and they are multidimensional, multidirectional, and multifunctional. We believe that taking a lifespan perspective has the potential to substantially advance our understanding of the development, and impact, and characteristics of VoA throughout life. We encourage more discussion of what a lifespan approach to VoA might look like and how it can inform and promote basic and applied research as well as public discourse and policies.
