# Correction to: Views on ageing: a lifespan perspective

**DOI:** 10.1007/s10433-021-00602-0

**Published:** 2021-02-17

**Authors:** Anna E. Kornadt, Eva‑Marie Kessler, Susanne Wurm, Catherine E. Bowen, Martina Gabrian, Verena Klusmann

**Affiliations:** 1 Fakultät für Psychologie und Sportwissenschaft, Differentielle Psychologie und Psychologische Diagnostik, Bielefeld University, Universitätsstraße 25, 33615 Bielefeld, Germany; 2 Department of Psychology, Geropsychology, MSB Medical School Berlin, Siemens Villa, Calandrellistraße 1-9, 12247 Berlin, Germany; 3 Institut für Community Medicine, Abt. für Sozialmedizin und Prävention, Universität Greifswald, Walter-Rathenau-Str. 48, 17475 Greifswald, Germany; 4 Independent Researcher, Vienna, Austria; 5 Independent Researcher, Frankfurt am Main, Germany; 6 Department of Psychology, Psychological Assessment and Health Psychology, University of Konstanz, P.O. Box 47, 78457 Constance, Germany; 7 Department of Psychology and Human Movement Science, Public Health, University of Hamburg, Mollerstr. 10, 20148 Hamburg, Germany

## Correction to: European Journal of Ageing (2020) 17:387–401 10.1007/s10433-019-00535-9

The article “Views on ageing: a lifespan perspective”, written by Anna E. Kornadt, Eva-Marie Kessler, Susanne Wurm, Catherine E. Bowen, Martina Gabrian and Verena Klusmann., was originally published electronically on the publisher’s internet portal on 11 October 2020 without open access. With the author(s)’ decision to opt for Open Choice, the copyright of the article changed on 05 January 2021 to © The Author(s) 2021 and the article is forthwith distributed under a Creative Commons Attribution 4.0 International License, which permits use, sharing, adaptation, distribution and reproduction in any medium or format, as long as you give appropriate credit to the original author(s) and the source, provide a link to the Creative Commons licence, and indicate if changes were made. The images or other third-party material in this article are included in the article’s Creative Commons licence, unless indicated otherwise in a credit line to the material. If material is not included in the article’s Creative Commons licence and your intended use is not permitted by statutory regulation or exceeds the permitted use, you will need to obtain permission directly from the copyright holder. To view a copy of this licence, visit http://creativecommons.org/licenses/by/4.0.

